# Mapping the complexity of motor variability: From individual space of variability to motor fingerprints

**DOI:** 10.3758/s13428-025-02635-0

**Published:** 2025-04-09

**Authors:** J. Manuello, T. Ciceri, V. Longatelli, C. Maronati, E. Biffi, A. Cavallo, L. Casartelli

**Affiliations:** 1 Move’N’Brains Lab, Department of Psychology, University of Turin, Turin, Italy; 2 NeuroImaging Lab, Scientific Institute IRCCS E. Medea, Bosisio Parini (LC), Italy; 3 Department of Information Engineering, University of Padua, Padua, Italy; 4 Theoretical and Cognitive Neuroscience Unit, Scientific Institute IRCCS E. Medea, Bosisio Parini (LC), Italy; 5 Bioengineering Lab, Scientific Institute IRCCS E. Medea, Bosisio Parini (LC), Italy

**Keywords:** Individual space of variability, Motor variability, Motor fingerprint, Procrustes transformation, Gait kinematics

## Abstract

Traditional average-based metrics have long been considered the gold standard in behavioral and brain research. However, recent advances emphasize the importance of examining the dispersion around the mean to uncover the nuances of individual differences and challenge simplistic assumptions. Thus, the study of *variability* is becoming increasingly central across a wide range of domains. Here, we tackle the composite architecture of *motor variability* by proposing a new geometric method to model it. Three independent gait datasets are used to: i) develop the method (Dataset 1), ii) evaluate its performance when transitioning from optoelectronic cameras to inertial measurement units (Dataset 2), and iii) generalize it in an experimental design with cognitive manipulations (Dataset 3). The method is based on the Procrustes transformation and multidimensional scaling. This geometric approach allows us to define the individual *space of variability* (i.e., the amount of bidimensional space covered by each individual’s trial-by-trial data). In turn, it provides robust evidence to identify each individual unique and specific motor signature (*motor fingerprint).* Our approach represents a fundamental shift from previous research: It is not the value of kinematic parameters *per se* that defines an individual’s motor signature, but rather the distinct way in which each individual *varies* these parameters, i.e., the dispersion of the distribution of their kinematic data. This novel perspective provides a single-subject-weighted proxy for motor signature, based on the characteristic dispersion of each individual’s data. The potential applications of this new method in research and clinical settings represent a fascinating future challenge.

**Supplementary Information:**

The online version contains supplementary material available at 10.3758/s13428-025-02635-0.

## Introduction

Recent advancements in theoretical, computational, and statistical methods have increasingly emphasized the importance of studying the data dispersion around the mean, i.e., *variability,* positioning it as a compelling and promising perspective for exploring human motor gestures. Indeed, the shift towards understanding variability has already provided essential insights into the complex, multivariate nature of human neurocognitive processes and behaviors (Genon et al., 2022; Loth et al., 2021). Such an approach is gradually overcoming the traditional scientific research predominantly focused on average-based metrics, which, while informative, often obscures the richness and diversity of individual differences.

The focus on variability, both *between* and *within* individuals, is particularly crucial in the context of motor behavior, where inconsistent findings—such as whether motor variability is beneficial or detrimental—highlight the complexity of the field (Dhawale et al., 2017; Todorov & Jordan, 2002).

Motor variability indeed presents a unique puzzle. On the one hand, it might seem trivial or irrelevant, as in the case of seemingly identical steps taken down a hallway. On the other hand, closer examination of actions on a trial-by-trial basis reveals a rich and complex architecture of variability (Casartelli et al., 2023). For example, even seemingly routine actions, like grasping a cup or executing a forehand shot in tennis, show movement kinematics that are never exactly replicated (Becchio et al., 2018; Ciceri et al., 2023; Ting et al., 2015). Although similar dualisms in philosophy are far from being considered a novelty, as noted by Heraclitus' adage that one never swims in the same river twice, two aspects deserve consideration: First, robust methods for quantitatively analyzing motor variability are relatively scarce; Second, there is a lack of theoretical frameworks that explore the functional implications of such analyses.

From a methodological point of view, given the multidimensional nature of kinematic data, modeling the (dis)similarity between movements or actions is a complex task that requires a balance between data simplification (i.e., dimensionality reduction) and the preservation of essential characteristics, such as the biomechanical properties of kinematic parameters.

Over the last decade, various approaches have been explored to quantitatively describe the relationships among motor events. In this context, Słowiński and colleagues (2016) used the earth mover’s distance (EMD) (Levina & Bickel, 2001) to compare hand velocity profiles of different subjects performing a motor task under various experimental conditions. They found that pairs of subjects with a smaller EMD were more likely to achieve coordination during interaction. However, this method, which models data through frequency histograms, focuses on a single kinematic variable. As a result, using a different kinematic variable could yield different results. Other commonly used methods, such as correlational analyses (De Marco et al., 2020) and dynamic time warping (DTW) (Sakoe & Chiba, 1978), also derive relevant measures of individuals’ kinematic profiles but are typically applied to one variable at a time. Thus, they fail to account for the multidimensionality of kinematic data and, even more critically, their use in the literature entirely overlooks the intra-individual trial-by-trial variability in movement kinematics. Thus, there remains a need for methods that can effectively model the full spectrum of kinematic features across multiple dimensions and capture the nuanced variability *within* an individual’s motor performance.

In this study, we aimed to address these gaps by proposing a geometric approach based on the Procrustes transformation and multidimensional scaling (MDS) technique to model (i.e., to quantify and represent) within-subject trial-by-trial motor variability (Phase I). This method allowed us to define the construct of individual *space of variability* (Phase II)*,* which in turn reveals the distinctive components to characterize the individual *motor fingerprint* (Phase III).

As it will be elaborated in the following sections, our choice of a geometric approach based on Procrustes transformation in combination with MDS was developed, evaluated, and generalized on three different gait datasets (Ciceri et al., 2023; Rengifo Rodas et al., 2020; Sharma et al., 2023). These datasets differ in terms of sample size, technical aspects, and (the presence of) cognitive manipulations, making each one particularly suitable for specific parts of the research.

Based on converging experimental evidence highlighting the unique ways individuals express motor (and even neural) patterns (Arazi et al., 2017; Cavallo et al., 2021; Lu et al., 2024; Vidal & Lacquaniti, 2021), we hypothesized that distinct individual-wise patterns of data distribution should emerge from the bidimensional representation of each individual’s kinematics. Thus, any individual-wise clustering defines a specific *space of variability* as a coherent, reliable indicator of the individual’s unique motor signature. Notably, the consistency in these patterns across different experimental sessions (Dataset 1 and Dataset 2) or manipulations (Dataset 3) underscores a *composite* yet *coherent* principle (Casartelli et al., 2023) that each individual’s space of variability is a reliable proxy for representing the architecture of their specific and unique motor signature, or “*motor fingerprint*.”

## Methods

The aim of this work was to develop, evaluate, and generalize a method to model (i.e., to quantify and represent) within-subject trial-by-trial variability (Phase I) and to define the individual space of variability (Phase II) to be used as a reliable proxy to determine the architecture of individuals’ motor fingerprint (Phase III). To do so, we used a set of kinematic variables of the lower limb extracted from gait cycles. Since gait occurs mainly in the sagittal plane with joint flexion/extension range of motion exhibiting higher variation, the following gait variables were computed for each step of each dataset, separately for the right and left legs:

- Angular velocity of the hip flexion/extension
- Angular velocity of the knee flexion/extension
- Angular velocity of the ankle flexion/extension

A step was defined as a complete stride cycle starting with the initial contact of the foot (either right or left) with the ground, marked by the heel strike, and concluding at the end of the swing phase, encompassing a full gait cycle.

The choice to consider movement velocities followed previous literature attempting to define an individual signature starting from kinematic features (Słowiński et al., 2016). In order to facilitate cross-step comparisons regardless of their actual duration, each time window corresponding to a single-leg gait cycle was segmented into 100 time points, from 1 to 100% of step time. Subsequently, a moving average with no overlap was computed over each decile, so that, for example, the value of angular velocity at the first time point represents the mean angular velocity measured during the first 10% of the movement time, and so on.

This same set of rules was followed for each of the three datasets considered for the analyses and described below.

### Datasets

#### Dataset 1: Development of the method

The first of the three datasets used in the present work had been previously acquired and described by Rengifo Rodas and colleagues (Caicedo et al., 2020; Rengifo Rodas et al., 2020). Briefly, this consists of 44 healthy participants (37 female, 7 male; age 70 ± 8.6 years) who performed five runs, each 12 m long, of overground self-paced walking (please note that hereafter we will consistently use “step” to refer to a single gait cycle, “run” to refer to a single experimental session consisting of multiple steps of one subject, and “instance” to refer to the combination of runs within a subject. When the dataset is composed of only two runs, each instance corresponds to a single run.) As described by the authors, the gait data were acquired through seven optoelectronic cameras (Vicon Vantage V5), set to a sampling frequency of 100 Hz, together with a montage of 24 reflective markers placed on legs and hips (Table 1). After data inspection, two participants had to be excluded due to data insufficiency. Therefore, only the remaining 42 were considered for the present research.

**Table 1:** Overview of the datasets analyzed

Authors	Participants analyzed	Whole dataset Mean age ± *SD*	Technical details	Instances per participant
Dataset 1: Rengifo Rodas et al., 2020	42	70 ± 8.6 years	Overground walking; optometric cameras	2
Dataset 2:Sharma et al., 2023	7	26.2 ± 2.7 years	Overground walking; IMUs	2
Dataset 3:Ciceri et al., 2023	31	27.1 ± 4.3 years	Treadmill walking; optometric cameras	5

This dataset was selected to develop the proposed method due to the presence of multiple runs for each participant. In fact, this allowed us to generate two instances for each participant, combining the first two and the last two available subjects’ runs. Since the original experimental procedure by Rengifo Rodas and colleagues established the sequential execution of five walking runs without any peculiar experimental manipulation, the motor variability exhibited cannot be attributed to the presence of experimental manipulations that could change over time. Moreover, as participants received the same instruction for the five runs, the two instances generated for each subject should a priori be expected to show minimal differences among them.

#### Dataset 2: Evaluation of the method

The second of the three datasets used in the present work had been previously acquired and described by Sharma and colleagues (2023). Briefly, this consists of 11 healthy participants (5 female, 6 male; age 26.2 ± 2.7 years) performing out-of-lab overground self-paced walking, while an MTw Awinda suit equipped with inertial measurement units (IMUs) was used to record kinematics with a sampling frequency of 60 Hz. In order to follow the approach designed for Dataset 1, two runs per participant were analyzed, each one lasting approximately 2.5 min. This criterion led to the exclusion of four participants for which only one run was available (Table 1).

This dataset was selected to evaluate the developed method in a markedly different experimental setting compared with Rengifo Rodas et al. First, gait data were acquired through different technologies (IMU vs. optometric cameras). Second, participants walked for a different time window (minutes vs. seconds). Third, the analyzed sample was much smaller (7 vs. 42 participants) and younger (mean age 26 vs. 70 years).

#### Dataset 3: Generalization of the method

The third and last dataset used in the present work had been previously acquired and described by Ciceri and colleagues (2023). Briefly, this consists of 32 healthy participants (16 female, 16 male; age 27.1 ± 4.3 years) walking at a self-paced speed on the dual-belt treadmill that equipped the Gait Real time Analysis Interactive Lab (GRAIL, Motek, Netherlands). Gait data were acquired by means of 10 optoelectronic cameras set to a sampling frequency of 100 Hz and a montage of 26 reflective markers to track the two legs and the trunk. The kinematic data acquired through the GRAIL system were de novo preprocessed for the present study using the Gait Offline Analysis Tool (GOAT). First, data were low-pass filtered with a second-order Butterworth filter with a cutoff frequency of 6 Hz. Then, gait events detection and step segmentation were based on the built-in features of the GOAT software, which are optimized for the processing of treadmill-based gait data (Table 1). After data inspection, one participant was excluded in the current work due to missing ankle information caused by marker falling that significantly influenced the new analyses proposed here.

This dataset was selected to generalize the developed method since the experimental design of the original study provided several well-controlled experimental manipulations. Indeed, the original study was based on the manipulation of two contrasting *scenarios* (i.e., “risky and potentially dangerous” scenario and “safe and comfortable” scenario) and two experimental *conditions* (“motor interference” condition and “motor expectation” condition). Each experimental condition started with a one-minute *baseline* walking period (i.e., without any influence of the scenarios). For the aim of the current work and for Occam’s razor, we state that each participant performed 10 (2 + 2 + 2 + 2 + 2) runs of a one-minute walk under five different “manipulations” of auditory stimulation: risky expectation (n.2), risky interference (n.2), safe expectation (n.2), safe interference (n.2), and baseline (n.2) without any auditory stimulation. Thus, for the sake of clarity, we stress that for generalizing our method, we benefited from the availability of multiple manipulations as critical factors of the model (i.e., they are sources of variability). However, we did not directly focus on the specific semantic or neurocognitive aspects related to Ciceri et al.’s manipulations being considered irrelevant for the development of the method. In brief, the presence of semantic or neurocognitive manipulations in the original experimental design (Ciceri et al., 2023) makes it particularly suitable for testing our current aims (i.e., to generalize a newly developed method that models the individual space of variability as a proxy for individuals’ motor fingerprints).

In line with the data processing adopted for Dataset 1, the two runs of the same manipulation (i.e., risky expectation, risky interference, safe expectation, safe interference, and baseline) were pooled together, to obtain five instances for each participant. Notably, this dramatically increased the complexity of the model, allowing the generalization of the method originally developed on two instances per participant in a design with five instances each, and accounting for thousands more steps.

### Quantification of individual* space of variability*

The developed procedure, from the preprocessed data to the quantification of the individual space of variability, can be divided into distinct phases: Phase I, computation of inter-event distance [I-a] and representation of the events in a lower-dimensional space [I-b], and Phase II, characterization of the individual space of variability. In general terms and for promoting clarity in the exposition, we can also rephrase the logical flow: to model—and therefore quantify and represent—an individual space of variability, it is preliminarily necessary to locate each specific event in the space [I-b]. This, in turn, requires first knowing the spatial relationships among the events [I-a].

#### Computation of inter-event distance: Phase [I-a]

Several approaches have been proposed in the literature to quantify the similarity between movements or actions (Anwary et al., 2019; De Marco et al., 2020; Guo et al., 2017; Słowiński et al., 2016). Most recently, we have described a method based on the Procrustes transformation (Manuello et al., 2024). Its main advantage over competing alternatives (e.g., DTW, Euclidean distance, EMD) is to allow for the conjoint modeling of multiple variables, therefore comparing the movements/actions in a more comprehensive and ecological way. This cannot be achieved even through the multivariate version of DTW, in which each variable is time-stretched individually and the various results then combined. In general terms, the Procrustes transformation aims to find the optimal combination of translation, scaling, and reflection to be applied to the “comparison matrix” to minimize its distance from the “target matrix” (Bookstein, 1992). The output of this process is a “transformed matrix,” being the closest possible match with the target matrix while preserving the original shape of the comparison matrix. The remaining distance between the transformed and target matrices is called the Procrustes distance, computed as the sum of squared differences between the corresponding points in the two distributions (for a more comprehensive description of the use of Procrustes transformation for the computation of inter-event distance based on kinematics variables, see Manuello et al., 2024).

The aim of this first stage was to describe the existing relationships among the events where each step executed by each of the participants was considered as a single event.^1^ In Dataset 1, this resulted in one array of 1051 steps to be used as the input for the Procrustes analysis. The size of the array was 5594 steps in Dataset 2 and 23,974 steps in Dataset 3. Initially, the first step was taken as the 10 × 3 “target matrix” (10 time points by 3 variables: angular velocity of the flexion–extension of (i) the hip, (ii) the knee, and (iii) the ankle), and Procrustes transformation was iteratively applied between the “target matrix” and each of the *N* – 1 other steps (i.e., the 10 × 3 “comparison matrix”). The same procedure was repeated until all the N steps were used as “target matrix”. The final output of this first stage was therefore a 1051 × 1051 (or 5594 × 5594, or 23,974 × 23,974) symmetric matrix, containing the Procrustes distance (in the range 0–1) between each possible pair of steps across all subjects. In the present work, the Procrustes transformation was used as implemented in the MATLAB function “procrustes,” allowing translation, scaling, and reflection.

#### Representation of the events in a lower-dimensional space: Phase [I-b]

The result of the computation of inter-event distance can be represented, in its original form, in a high-dimensional space. However, such a complex space would dramatically hinder visual interpretation of the data. A practical strategy in comparable situations consists in employing dimensionality reduction, a class of powerful mathematical procedures designed to decrease the complexity of a dataset. These procedures aim to condense the data into a more manageable form that facilitates comprehension and analysis, ensuring that the essence and integrity of the information are preserved as much as possible. Among many available techniques, MDS was deemed to be the most appropriate. In fact, it builds a lower-dimensional space, preserving the distances between points (in this case the inter-event distance between couples of steps) as closely as possible to those originally computed in the high-dimensional space (Torgerson, 1958). A crucial aspect of MDS is the selection of the number of dimensions to be used to build the representational space. In order to preserve the integrity of the information maximizing the interpretability of the results, the MDS was set to two dimensions.

#### Definition of the space of variability: Phase [II]

**Fig. 1:**
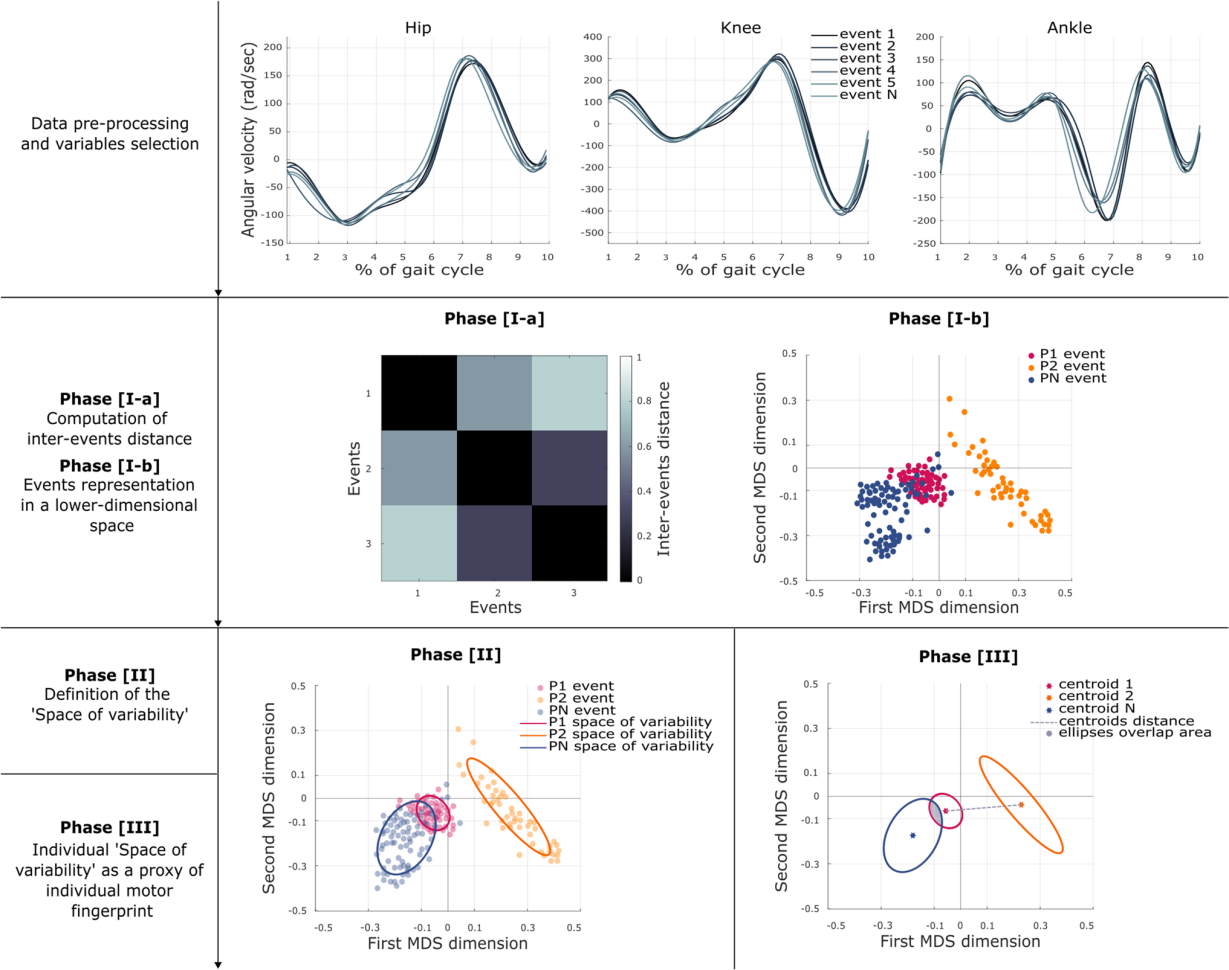
Graphical overview of the methodological pipeline. The developed procedure starts with data preprocessing and variables selection (upper panels). The quantification of the individual space of variability is divided into distinct phases. Phase I: Computation of inter-event distance [I-a] and representation of the events in a lower-dimensional space [I-b] (central panels). Phase II: Characterization of the individual space of variability (lower-left panel). The quantification of the individual space of variability is used as a proxy for determining the individual motor fingerprint (Phase III, lower-right panel). Please note that in the graphical representation, each event corresponds to a participant’s step

Finally, the obtained bidimensional representation was used to compute the individual *space of motor variability*. Here, this was operationalized as the amount of the bidimensional space covered by the steps walked by a given subject in each instance. In these terms, the wider the space, the greater the variability of execution. Specifically, an ellipse was determined by fitting a bivariate Gaussian distribution to the coordinates of the considered steps. Their mean values determined the ellipse center, while the eigenvectors of the covariance matrix indicated the major and minor axis directions. The lengths of these axes for a covariance ellipse containing the desired probability mass were determined by the square roots of the eigenvalues of the covariance matrix multiplied by the Mahalanobis radius, equal to 0.7 (Słowiński et al., 2016). According to the number of instances taken into consideration, two ellipses were generated for each subject in Dataset 1 and Dataset 2, while five ellipses were generated for each subject in Dataset 3 (Fig. 1).

### Individual space of variability as a proxy for individual motor fingerprint: Phase [III]

The methods described have allowed us to quantitatively capture the variability displayed by participants across various instances, resulting in what we term the individual space of variability. We propose that this space can serve as a reliable proxy for each subject’s unique motor fingerprint. Specifically, we designate the individual motor fingerprint as the total area encompassed by each participant’s multiple ellipses, representing different instances across Datasets 1 and 2, and varying experimental manipulations in Dataset 3 (i.e., risky expectation, risky interference, safe expectation, safe interference, and baseline). The construct of the individual motor fingerprint in Dataset 3 marks a significant advancement compared to those in Datasets 1 and 2. It comprehensively incorporates multiple experimental manipulations, allowing us to examine an individual’s motor behavior across diverse scenarios rather than isolating each condition. This broader approach ensures that the motor fingerprint captures an individual’s consistent motor patterns across varying situations, thereby reflecting a principle of regularity unique to each subject. Finally, and even more crucially considering that this point is the main novelty of our method and it takes all three datasets into account, such a construct of motor fingerprint is based on each individual's specific and unique motor signature in exploring (or spreading into) the space (i.e., trial-by-trial variability).

#### Distance between centroids

To investigate the construct of space of variability as a proxy for an individual’s motor fingerprint, the relationships among the ellipses were assessed based on their centroids. Specifically, if the proposed method accurately estimates an individual’s fingerprint, the average distance between the centroids of the ellipses representing a participant’s different instances (i.e., intra-subject distance) should be smaller than the average distance between these centroids and those of the ellipses representing other participants’ instances (i.e., inter-subject distance). To test this, the Euclidean distance between each possible pair of centroids was calculated, and a paired-sample *t*-test was conducted to compare the mean intra-subject and mean inter-subject distances. In addition, the percentage of cases in which the intra-subject distance was lower than the inter-subject distance was computed. To be clear, we use the term “I-self” to refer to the similarity between distinct instances of the same subject (i.e., intra-subject distance), and “I-others” to refer to the similarity among instances of different subjects (i.e., inter-subject distance; for a comparable approach, see Troisi Lopez et al., 2022).

#### Area of overlap between ellipses

While centroids are efficient descriptors for investigating the reciprocal positions of the ellipses, they do not really contain a measure of individual variability. In fact, infinite ellipses with different area could be built around a centroid. To overcome this issue, the same rationale explained above was investigated considering the area of overlap between the ellipses. In this case, if the proposed method correctly estimates an individual motor fingerprint, the mean area of overlap among the (two or five) ellipses describing a subject under the different instances (i.e., intra-subject overlap) should be greater than the mean area of overlap among those (two or five) ellipses and the (two or five) ellipses describing each other subject (i.e., inter-subject overlap). A paired sample *t*-test was therefore used to verify the inequality between mean intra-subject (I-self) area of overlap and mean inter-subject (I-others) area of overlap. In addition, the percentage of cases in which the intra-subject overlap was greater than the inter-subject overlap was computed.

### Control analyses

In order to gain a deeper understanding of the functioning of the ellipse-based approach and to compare it with a known metric, the areas of the obtained ellipses were correlated with the averaged standard deviation of the three kinematic variables considered for the analyses. Positive values of Pearson’s correlation would confirm that subjects exhibiting a greater dispersion of velocity profiles are also described through a wider ellipse area, meaning a wider space of variability. The same analysis was also replicated substituting in the pipeline the Procrustes algorithm with DTW, to provide a comparison with the most widely used method for time series transformation (see Supplementary Information).

## Results

### Space of variability

**Fig. 2:**
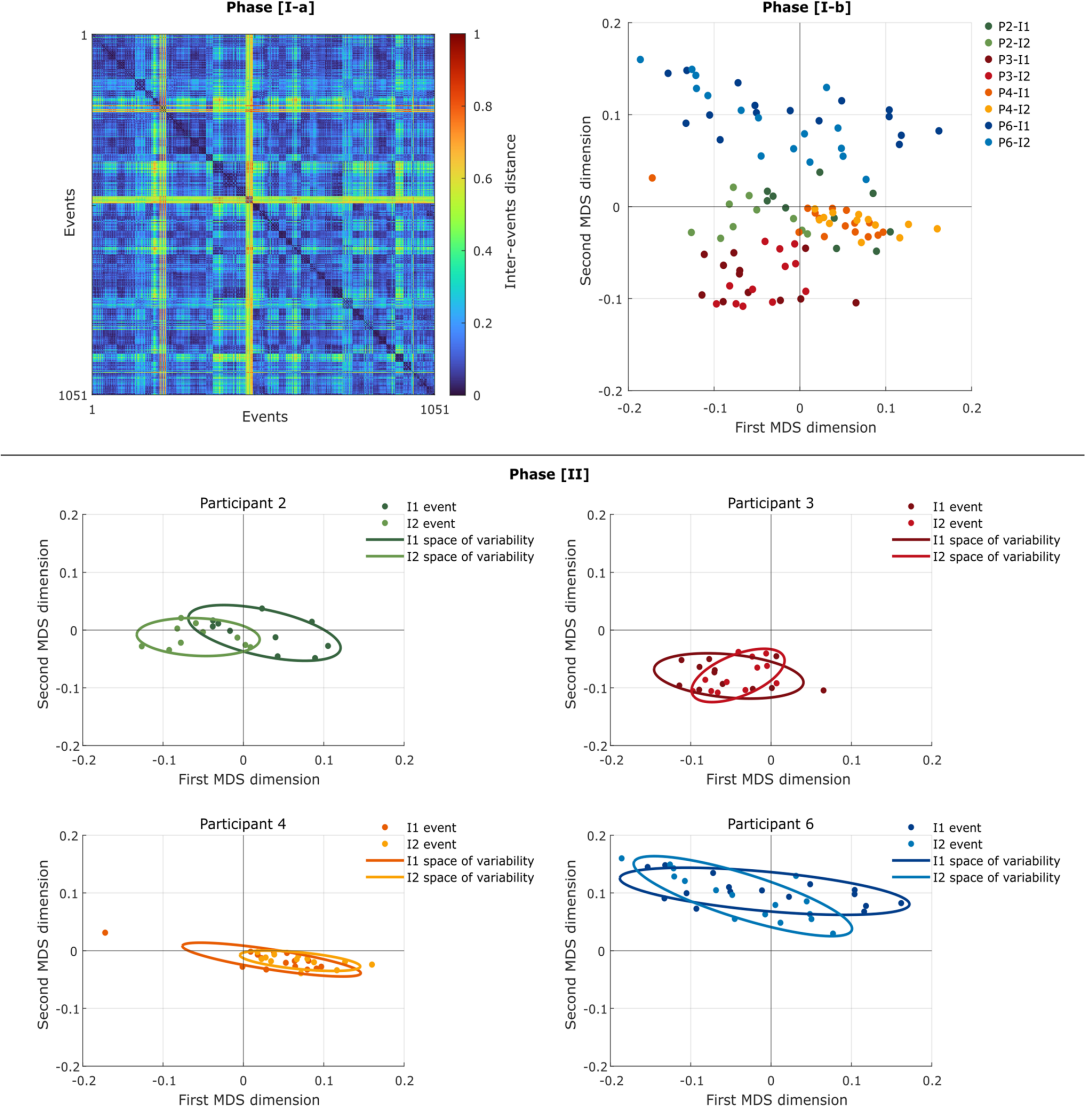
Top left: The inter-event motor distance as computed through Procrustes transformation on Dataset 1. Each row (and each column) represents one of the 1051 steps. Top right: representation of a subset of the events of Dataset 1 in a two-dimensional space after dimensionality reduction. Each circle represents a step, and steps of the same color belong to a same instance. For visualization purposes, participants were selected based on the clarity of the representation of their data. Bottom: The geometric representation of the space of variability of the participants selected above. Each circle represents a step. Note that although the participants are represented separately, the position of the points derives from the MDS computed on the whole 1051 steps, and therefore conveys the information of each other. P = participant, I = instance. The corresponding information for Dataset 2 and Dataset 3 is reported in the Supplementary Information

Phase [I-a] of the computational procedure (i.e. “computation of inter-event distance”) generated a 1051 × 1051 symmetric matrix for Dataset 1 (Fig. 2, top left), a 5594 × 5594 symmetric matrix for Dataset 2 (Fig. S1), and a 23,974 × 23,974 symmetric matrix for Dataset 3 (Fig. S3).

The maximum measured Procrustes distances were 0.908 for Dataset 1, 0.544 for Dataset 2, and 0.952 for Dataset 3. Starting from the Procrustes matrix, Phase [I-b] (i.e., “representation of the events in a lower-dimensional space”) generated a two-dimensional MDS, locating the steps comprehensively walked by the subjects (separately for each dataset) in any possible instances (Fig. 2, top right).

Finally, Phase II (i.e., “definition of the space of variability”) computed and projected the ellipses on top on the MDS (Fig. 2, bottom).

### Motor fingerprint

#### Distance between centroids

For Dataset 1 (i.e., overground walking, optoelectronics cameras, two instances per participant), the average intra-subject distance (i.e., I-self) measured on the whole sample was 0.038 ± 0.030. The average inter-subject distance (i.e., I-others) measured on the whole sample was 0.163 ± 0.052. The paired-sample *t*-test confirmed that the two distances were significantly different (*t*_41_ = 13.585; *p* < 0.001). Coherently, the inter-subject distance was found to be greater than the intra-subject distance in 93% of the cases (Table 2).

**Table 2:** Synopsis of the results for the three datasets. All differences between intra-subject and inter-subject values were statistically significant

		Centroids distance			Area overlap	
	**Intra-subject**	**Inter-subject**	**Percentage**	**Intra-subject**	**Inter-subject**	**Percentage**
**Dataset 1**	0.038 ± 0.030	0.163 ± 0.052	93%	0.406 ± 0.130	0.072 ± 0.038	96%
**Dataset 2**	0.018 ± 0.013	0.067 ± 0.014	89%	0.559 ± 0.190	0.196 ± 0.083	90%
**Dataset 3**	0.053 ± 0.039	0.127 ± 0.042	89%	0.484 ± 0.147	0.136 ± 0.058	98%

Moving to Dataset 2 (i.e., overground walking, IMU, two instances per participant), the average intra-subject distance (i.e., I-self) measured on the whole sample was 0.018 ± 0.013. The average inter-subject distance (i.e., I-others) measured on the whole sample was 0.067 ± 0.014. The paired-sample *t*-test confirmed that the two distances were significantly different (*t*_6_ = 6.061; *p* < 0.001). Coherently, the inter-subject distance was found to be greater than the intra-subject distance in 89% of the cases (Table 2).

Finally, when considering Dataset 3 (i.e., treadmill walking, optoelectronic cameras, five instances per participant), the average intra-subject distance (i.e., I-self) measured on the whole sample was 0.053 ± 0.039. The average inter-subject distance (i.e., I-others) measured on the whole sample was 0.127 ± 0.042. The paired-sample *t*-test confirmed that the two distances were significantly different (*t*_30_ = 10.224; *p* < 0.001). Coherently, the inter-subject distance was found to be greater than the intra-subject distance in 89% of the cases (Table 2).

#### Area of overlap between ellipses

When the areas of overlap between ellipses were considered, for Dataset 1 the average intra-subject (I-self) overlap measured on the whole sample was 0.406 ± 0.130, while the average inter-subject (I-others) overlap was 0.072 ± 0.038. The paired-sample *t*-test confirmed that the two overlap areas were significantly different (*t*_41_ = 15.960; *p* < 0.001). Coherently, the intra-subject overlap was found to be greater than the inter-subject overlap in 96% of the cases (Table 2).

In Dataset 2, the average intra-subject (I-self) overlap measured on the whole sample was 0.559 ± 0.190, while the average inter-subject (I-others) overlap was 0.196 ± 0.083. The paired-sample *t*-test confirmed that the two overlap areas were significantly different (*t*_6_ = 4.2095; *p* < 0.01). Coherently, the intra-subject overlap was found to be greater than the inter-subject overlap in 90% of the cases (Table 2).

Finally, when considering Dataset 3, the average intra-subject (I-self) overlap measured on the whole sample was 0.484 ± 0.147, while the average inter-subject (I-others) overlap was 0.136 ± 0.058. The paired-sample *t*-test confirmed that the two overlap areas were significantly different (*t*_30_ = 13.772; *p* < 0.001). Coherently, the intra-subject overlap was found to be greater than the inter-subject overlap in 98% of the cases (Table 2).

### Control analyses

The average Pearson's correlation between the ellipses’ areas and the averaged standard deviation of the three kinematic variables considered for the analyses were statistically significant (all *p*_s_ < 0.001). Pearson’s *r* was 0.84 for Dataset 1, 0.90 for Dataset 2, and 0.73 for Dataset 3 (average *r* = 0.82). For DTW results, please see Supplementary Information.

## Discussion

Motor variability is a complex, multilayered construct that encompasses different levels of description, heterogeneous degrees of internal coherence, and inconsistent operational uses. From a theoretical point of view, we have recently addressed the controversial use of cognate terminologies (e.g., variation, noise, adjustments, fluctuations) by providing a theoretical model that deconstructs the monolithic view of motor variability (Casartelli et al., 2023). The fundamental premise is that variability is neither simplistically beneficial nor detrimental. Instead, the key point is that studying motor variability is more an epistemological challenge than an ontological one (Uddin, 2020). This means that while one issue lies in operationalizing motor variability, another essential aspect is the conceptualization of the distinct factors that contribute to modulating specific subsets of motor variability, for example, modifying or adjusting one’s own motor responses due to environmental demands or due to the need to learn new motor skills (Casartelli et al., 2023). To illustrate this, consider the analogy with signal–noise theory. Denoising an electroencephalography (EEG) oscillatory signal is technically challenging because one must apply complex statistical and computational procedures. However, noise and signal in the EEG oscillatory domain are not fixed, universal concepts; they are *specific*, task-dependent, and even theory-dependent constructs (Cavallo & Casartelli, 2023). Thus, to denoise data we should have a clear conceptualization of signal/noise in that specific experimental scenario. As further proof of this, classical assumptions have stated that any intrinsic or non-evoked brain activity should basically be considered as irrelevant noise, but multiple convergent lines of evidence clearly confute this (Lisberger & Medina, 2015; Pezzulo et al., 2021; VanRullen, 2016). Similarly, we believe and state that the study of motor variability contains a comparable challenge.

While theoretical modeling of motor variability is crucial (Criscuolo et al., 2024; Haar, 2024; Panzeri & Nili, 2024; Vescovo & D’Ausilio, 2024), the operationalization of any theoretical model requires a rigorous methodology. Thus, in the present work we propose a new method that quantifies and represents within-subject motor variability, leading to the characterization of an individual space of variability.

The geometric approach we propose, based on the Procrustes transformation and MDS, is designed to parametrize within-subject trial-by-trial variability. The primary outcome of this method is the demonstration that it is possible to define each individual *space of variability* (Fig. 2, Figs. S2 and S4). Results showed in fact that intra-subject instances (i.e., ellipses) were significantly closer and more overlapping than inter-subject ones (Table 2). This remained true even in the case of Dataset 3, where the experimental manipulations induced a potentially greater intra-subject variability. Notably, Ciceri et al. had previously shown on the same dataset that the cognitive manipulation implemented was strong enough to induce biomechanical differences detectable by machine learning algorithms. Despite this, the method we proposed still identified consistent individual motor fingerprints. Analysis of the three datasets revealed that the individual space of variability could be reliably determined regardless of the number of subjects or steps performed, with a range spanning from 1051 to 23,974 total gait cycles. Interestingly, Dataset 2 contained approximately five times more steps overall than Dataset 1, despite having nearly one-sixth the participants, proving the method’s robustness across different participant-to-step ratios. Having said this, a question could arise concerning the minimum amount of information (i.e., number of steps, runs, or instances) necessary to attain a “complete” (or at least complete enough) fingerprint—in other words, how many steps should the subject walk to ensure that their full space of motor variability has been covered. There is probably no straightforward answer to this, and this is the reason that the quantified individual space of variability was presented as a “proxy” of individual motor fingerprints. In this acceptation, the construct of “motor fingerprint” should not be seen as a rigid “space” that should be clearly circumscribed, but rather as a mathematical or epistemological limit. Therefore, the proposed method is (at least) able to provide a compelling, robust, stable, replicable *proxy* for individuals’ motor fingerprints.

The method we propose also proved to be reliable for gait data collected using different motion capture techniques, from optoelectronic systems to IMUs. Interestingly, the maximum inter-step distance computed by Procrustes for Dataset 2, where IMUs were used (0.544), was approximately half of the maximum inter-step distance computed for the remaining datasets (0.908 for Dataset 1, and 0.952 for Dataset 2), based on optoelectronic cameras. It is noteworthy that IMU data are generally noisier than those from optoelectronic systems, yet our method still successfully identified clear intra-subject and inter-subject distinctions. Moreover, the combination of Procrustes and MDS accurately mapped the variability in the kinematic variables used as inputs, with the mean correlation between ellipse areas and the average standard deviation of angular velocities being high (*r* = 0.82) and statistically significant across the three datasets. Notably, when Procrustes transformation was replaced with DTW, the average correlation decreased to *r* = 0.78. Thus, our approach outperforms the widely used method for time series analyses and, even more importantly, offers the crucial advantage of simultaneously modeling multiple kinematic variables.

Beyond computational aspects, the space of variability obtained through our method both provides a straightforward, two-dimensional graphical representation of extensive data, and largely preserves the original biomechanical features of kinematic parameters (Manuello et al., 2024). This graphical representation facilitates intuitive exploration of the data (e.g., showing that one subject varies more than others) and suggests that each participant has a highly specific and unique *motor fingerprint.* In a broader sense, the idea that everyone has a specific and unique motor signature that is clearly recognizable across time or tasks is not completely new. Other scholars have explored this issue in the last decade (Cavallo et al., 2021; Vidal & Lacquaniti, 2021). However, our approach marks a fundamental shift: it is not the value of kinematic parameters *per se* that constitutes the architecture of one individual’s motor signature. Our approach shows a different but fundamental point: it is the specific and unique way through which one individual *varies* the scattering of events—i.e., the area covered by the distribution of their singular steps—that constitutes the architecture of one individual’s motor fingerprint. More simply, the specific and unique way an individual disperses data within the space of variability constitutes their motor fingerprint. This novel perspective provides a subtle, single-subject-weighted proxy for motor signature, grounded in each individual’s distinctive data dispersion.

The possibility of a unique motor signature deriving from individual variability opens up new and intriguing avenues for research and clinical applications. For instance, it challenges the reliance on case versus control studies and, more generally, on average-based metrics (Loth et al., 2021). Our approach advances beyond many methodologies that, while establishing individual differences, often restrict themselves to certifying or describing the idiosyncratic nature of each person at either a behavioral (e.g., motor) or neural (e.g., functional connectivity, see Lu et al., 2024) level. In contrast, our approach is expressly developed to face the inter-individual distances between the specific and unique individuals’ motor fingerprints, providing the essential starting point for any endeavor aiming at taking advantage of knowing inter-individual motor distances. How such an approach may be profitably employed in research, clinical and, notably, in rehabilitative settings is a matter of future intriguing and thought-provoking challenges.

### Limitations, future directions and conclusive considerations

Our geometric approach based on the Procrustes transformation and MDS (Phase I) proved to be robust in modeling within subject trial-by-trial motor variability. This method allowed us to define the architecture of individual space of variability (Phase II), which in turn provided the foundation for disclosing each individual’s motor fingerprint (Phase III). Although the combined use of multiple datasets allowed an in-depth characterization of gait, this obviously does not complete the entire spectrum of human gestures. Other movements, such as the non-cyclical ones, could entail more variable repetitions, possibly resulting in less-defined motor fingerprints. Nevertheless, Manuello et al. (2024) recently showed that the combination of Procrustes transformation and MDS can be effectively applied to completely different motor gestures, and there are no theoretical issues preventing the adaptation of the pipeline proposed here to any kind of (non)human motor performance.

Our approach fits nicely with recent trends in the literature that strongly emphasize the need for going beyond average-based metrics, stressing the importance of using data weighted on individual thresholds or baselines. In addition, it is relevant because it provides a robust metric to quantify intra- and inter-individual motor distances. Notably, the fact that any unique motor signature is grounded on how each individual specifically varies data (i.e., each individual’s distinctive way of dispersing data in the space) represents the main novelty.

A note of caution is nevertheless necessary for what concerns the analytical and quantitative interpretation of the defined space of variability. Any parameter concerning the geometry of ellipses is in fact strongly dependent on the solution found by the MDS, being therefore influenced by the specific set of steps (and obviously, subjects) considered. Consequently, even the insertion of a new step could slightly alter the final result. Therefore, as a practical example, the total area of each ellipse can be legitimately analyzed to infer which subjects express greater motor variability. But it should be kept in mind that if the area of subject A is equal to 10 and the area of subject B is equal to 3, subject A will surely be more variable than subject B, even after the inclusion of subject C in the sample, but the actual area values for subjects A and B will likely be different now.

More generally, although the geometric representation of motor variability provides a robust framework for analyses conducted within a study (i.e., *within-study* analyses), it is worth noting that the resulting values of each space of variability (e.g., the sizes of the ellipses) depend on the specific dataset used to compute them. Numerical comparisons between independent studies should therefore be made with caution. However, when raw data from different datasets relating to a task are available, direct comparisons become technically possible. In this scenario, the application of the same Procrustes transformation and MDS mapping to all datasets ensures that the distance matrices and the resulting motor fingerprints are comparable and interpretable across datasets. This approach enables researchers to generalize the method beyond individual studies and thus permits *between-study* comparisons, which can potentially offer a more complete picture of motor fingerprints across populations (e.g., clinical vs. nonclinical), measurement systems, and experimental manipulations. Where more standardization is required, strategies such as normalizing variability measures to a reference dataset or creating a shared embedding space for motor fingerprints through meta-analytical methods can be explored.

Lastly, it is important to note that while our focus has been on *motor variability*, the concept of variability extends to neural domains as well. Other critical aspects include *neural variability*, the *space of neural variability*, and the *neural fingerprint* (Arazi et al., 2017; Lu et al., 2024). A particularly intriguing future challenge is to explore potential covariation effects between neural and motor variability (for an elegant and pioneering study, see Haar et al., 2017; see also Casartelli et al., 2023; Panzeri & Nili, 2024). These investigations would align with the integrative framework that dynamically combines factors shaping the composite brain-body-environment system (Criscuolo et al., 2024). How these insights will be operationalized in the near future represents a stimulating and promising area of research.

## Supplementary Information

Below is the link to the electronic supplementary material.

 Supplementary Information (PDF 763 KB)

## Data Availability

The database used for the analyses presented is freely available from: Dataset 1: https://data.mendeley.com/datasets/xzv6bjm9x2/1 Dataset 2: 10.6084/m9.figshare.c.6076607.v1 Dataset 3: https://zenodo.org/records/8139385

## Footnotes

1 Please note that in this work the terms “event” and “step” are used interchangeably. In general, "event" is more appropriate because it preserves the possibility to apply the method to other datasets as well (e.g., with upper limb actions). In the context of this work, "step" is used because it clearly simplifies the storytelling.

## Code availability

The code used for the analyses presented can be downloaded from https://github.com/Jordi-Manuello/space_of_variability

## Declarations

### Open practice statement

Data and code are made freely available for other colleagues.
