# Supplementary material for: Mapping the complexity of motor variability: From individual space of variability to motor fingerprints
Source: Behav Res Methods. 2025 Apr 9;57(5):140. doi: 10.3758/s13428-025-02635-0 (PMC11982147; doi:10.3758/s13428-025-02635-0)
Supplement: Supplementary file 1 — Supplementary Information (PDF 763 KB) [file 13428_2025_2635_MOESM1_ESM.pdf]

Mapping the complexity of motor variability: from individual space of variability to motor fingerprints

Manuello J., Ciceri T., Longatelli V., Maronati C., Biffi E., Cavallo A.*°, Casartelli L°.

* Corresponding Author: Andrea Cavallo, andrea.cavallo@unito.it

° Co-last authorship

Supplementary Information

1. Comparison between Procrustes transformation and Dynamic Time Warping

As mentioned in section “Control analyses” of the main text, the obtained ellipses’ areas were correlated with the averaged standard deviation (std) of the 3 kinematic variables considered for the analyses. Based on the implemented pipeline, the ellipses’ areas depend on the results of MDS, that in turn was based on the motor distances computed through Procrustes transformation. While the Procrustes algorithm has been known for a long time, it was only recently proposed as a way to compute distance between kinematic profiles. Therefore, the performance of this method was compared with that of Dynamic Time Warping (DTW), being a reference for manipulation of time series in the field of human kinematics and human behavior research. In practical terms this means that after data preprocessing and quality assessment, the distance between any possible couple of steps was now computed with DTW. Notably, contrary to Procrustes transformation, DTW can work on a single kinematic variable at a time. This means therefore that the inter-step distance was computed 3 times, once for the angular velocity of hip flexion/extension, once for the angular velocity of knee flexion/extension, once for the angular velocity of ankle flexion/extension. The 3 obtained distance matrices where then averaged, and the resulting matrix was normalized between 0 and 1 to allow comparison with results based on Procrustes transformation. The mean and normalized DTW distance matrix was used as input for MDS, and the rest of the pipeline remained unchanged.

Results showed that the Pearson correlation between ellipses’ areas and std of the kinematic variables averaged over the three datasets was r=0.78.

2. Graphical representation of the results for Dataset 2

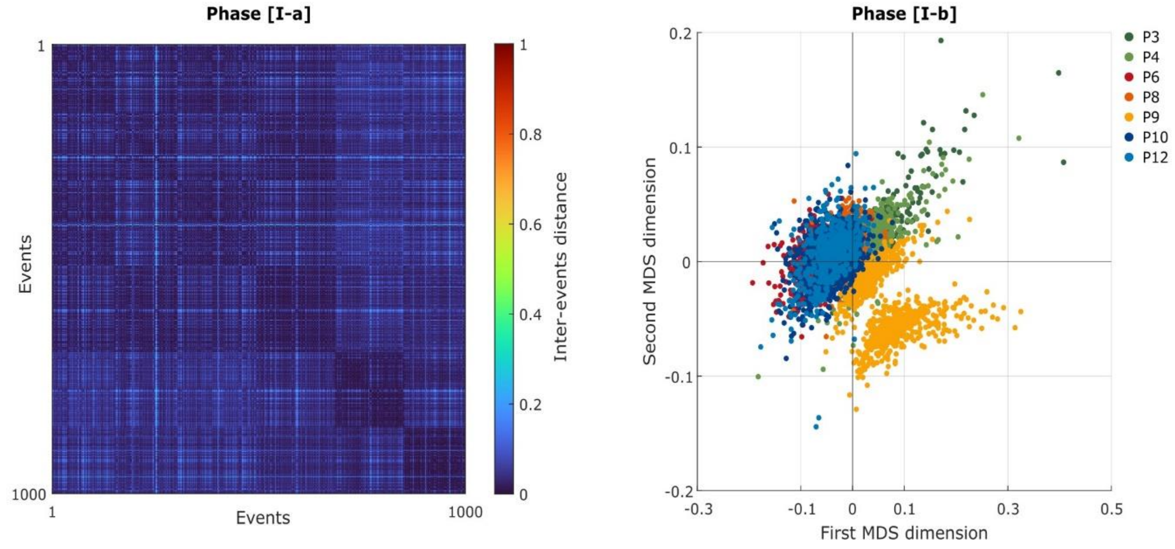

Figure S1: Left panel: The inter-events motor distance as computed through Procrustes transformation on Dataset 2. Each row (and each column) represents one step. Note that to allow visualization only the first 1000 rows and columns were represented. Right panel: Representation of the whole set of the events of Dataset 2 in a 2-dimensional space after dimensionality reduction. Each circle represents a step, and steps of the same color belong to steps of the same participant.

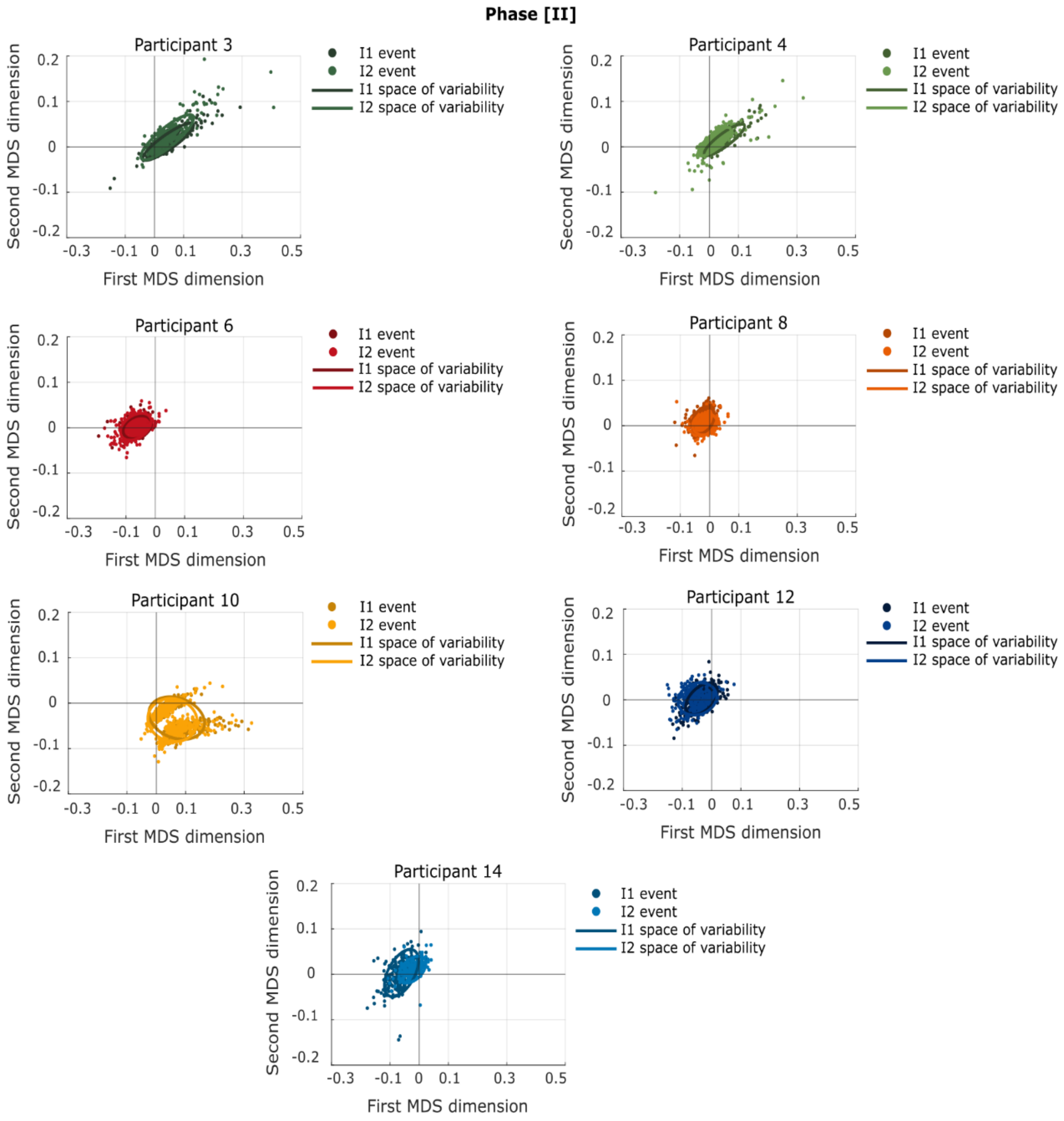

Figure S2: The geometric representation of the space of variability of each participant in Dataset 2. Each circle represents a step. Note that although the participants are represented separately, the position of the points derives from the MDS computed on the whole 5594 steps, and therefore convey the information of each other.

3. Graphical representation of the results for Dataset 3

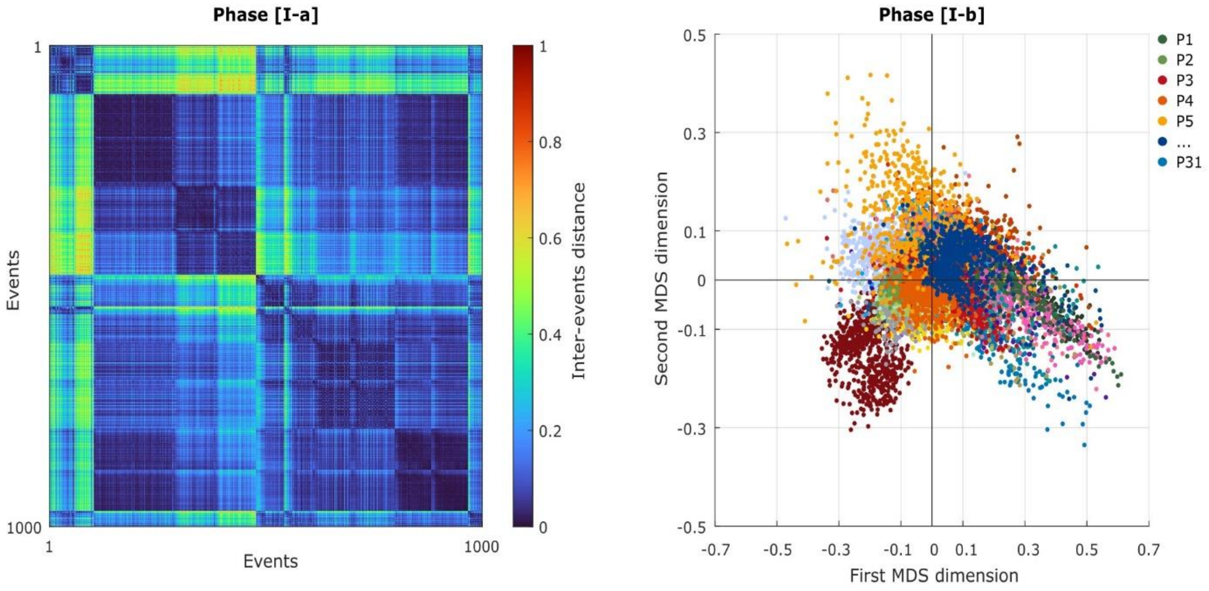

Figure S3: Left panel: The inter-events motor distance as computed through Procrustes transformation on Dataset 3. Each row (and each column) represents one step. Note that to allow visualization only the first 1000 rows and columns were represented. Right panel: Representation of the whole set of the events of Dataset 3 in a 2-dimensional space after dimensionality reduction. Each circle represents a step, and steps of the same color belong to the same participant.

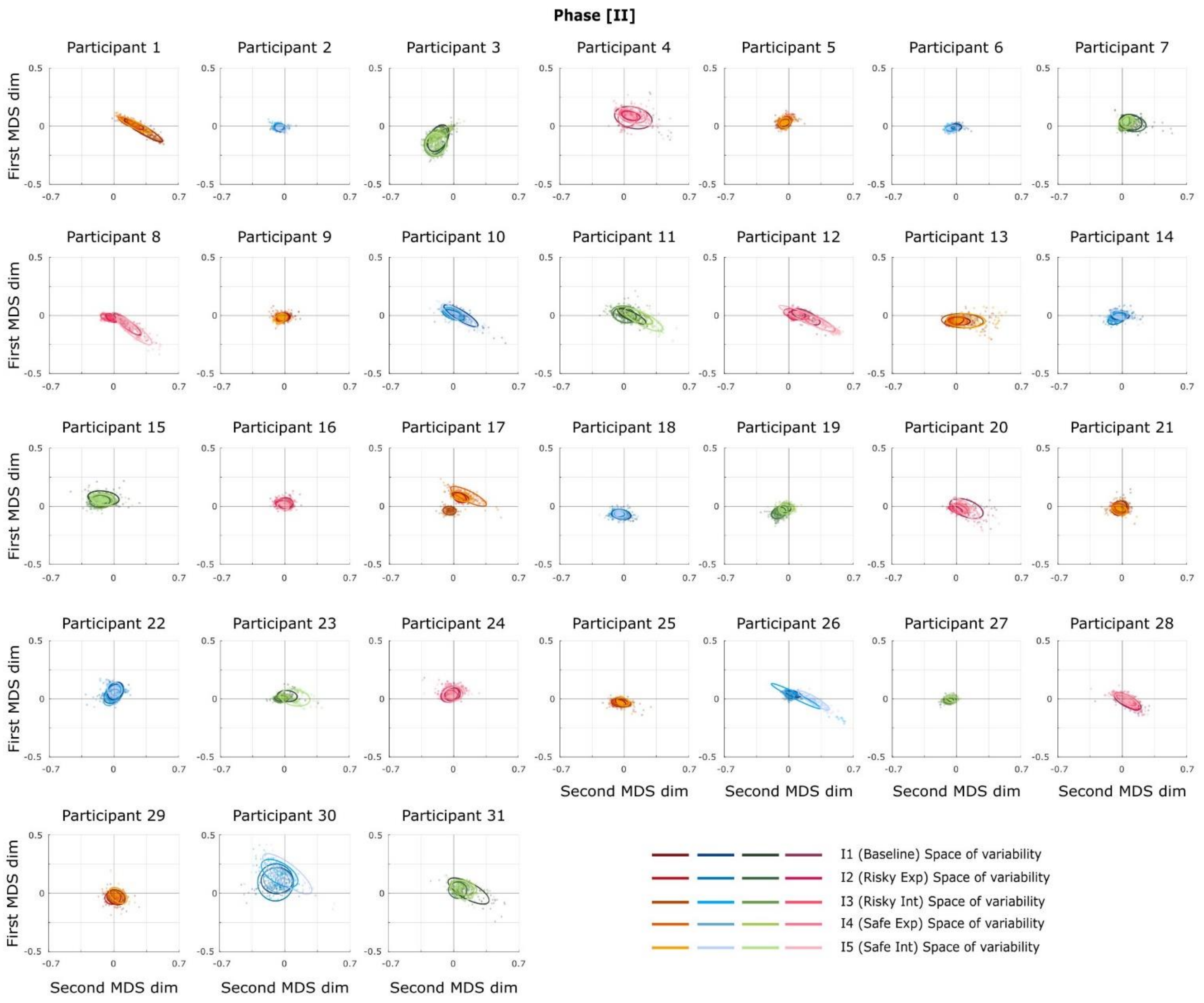

Figure S4: The geometric representation of the space of variability of each participant in Dataset 3. Each circle represents a step. Note that although the participants are represented separately, the position of the points derives from the MDS computed on the whole 23974 steps, and therefore convey the information of each other.
